# Analysis of the Evolution of Tannic Acid Stabilized Gold Nanoparticles Using Mie Theory

**DOI:** 10.1155/2014/832657

**Published:** 2014-11-23

**Authors:** Assia Rachida Senoudi, Sidi Mohammed Chabane Sari, Ilhem Faiza Hakem

**Affiliations:** ^1^Physics Department, University of Abou Bekr Belkaid, 13000 Tlemcen, Algeria; ^2^Chemistry Department, University of Abou Bekr Belkaid, 13000 Tlemcen, Algeria; ^3^Materials Science and Engineering Department, Carnegie Mellon University, 5000 Forbes Avenue, Pittsburgh, PA 15243, USA

## Abstract

Spherical gold nanoparticles (GNPs) have been synthesized in aqueous solutions using sodium citrate (SC) and tannic acid (TA) as reducing and stabilizing agents. Upon addition of TA and compared to the GNP TA-free aqueous solutions, a reduction of the GNPs size and consequently a dramatic change of their optical properties have been observed and quantitatively analyzed using Mie theory. An increase in the concentration of TA reveals a modification of the colloidal solution refractive index that is evidenced by the shift in the peak position of the localized surface plasmon resonance (LSPR) band. The variations of the peak absorbance with the TA concentration are examined in the low and high concentration regimes.

## 1. Introduction

The optical properties of gold nanoparticles (GNPs) of 2 nm to 100 nm in diameter have been the subject of intensive studies from both theoretical and experimental point of view due to their unique properties that offer new opportunities for technological applications [1–20]. Widely considered to be “bioinert” materials, gold particles have been suggested as candidate materials in diagnostic and therapeutic applications. They have also been considered as model systems for the fundamental studies on the role of spatial confinement on physical properties because of the abundance of chemical procedures to control size and shape of the gold nanoparticles.

The relevance of gold nanoparticles across a wide range of areas such as the biomedical field has rendered the development of “biocompatible” synthetic techniques a particularly important task. Traditional approaches for preparing stable gold colloids involve the reduction of the relevant metal salt in the presence of a stabilizer [9, 10, 21–27]. Conventional stabilizer systems have been based on organic surfactant systems (often applying third-coupling chemistry) that present a concern for biomedical applications and hence the development of “benign” stabilizers is of significant importance. One example that has attracted particular attention is the use of tannic acid (TA); however, the control of particle morphologies was shown to be very difficult with reactions leading to disperse products. For a more detailed discussion of the mechanism of TA-induced gold particle synthesis, the reader is referred to [2–5, 16–21].

The necessity to better control particle size in TA-mediated gold particle synthesis presents a challenge to analytical techniques to in situ monitor the size and shape during the synthetic process. Optical absorption spectroscopy (UV/vis) has been among the most widely used techniques to analyze the size and shape of gold nanoparticle products. UV/vis analysis is particularly sensitive method for the analysis of gold nanoparticles due to the strong plasmon resonance of metals and the sensitive dependence of the surface plasmon on the particle geometry. To relate the optical absorption spectrum to the particle geometry, the measured plasmon resonance has to be compared to model calculations. However, in all reported studies to date on TA-based systems (to the best of the authors' knowledge), these comparisons have been based on the so-called dipolar approximations in which the particle's absorption (and scattering) cross section is calculated based on only the leading order term of the polarization of the particle. While this approximation is generally valid for very small particles, it fails for larger particle systems (with *D* greater than about 20 nm) and hence the dipolar approximation provides only very limited information about the composition in size disperse particle systems such as TA-based gold nanoparticle products.

In this contribution we apply the full Mie theory [14] to the in situ analysis of particle size evolution in particles synthesized by TA reduction and stabilization. We demonstrate that Mie theory accurately captures the optical characteristics of particle systems across the whole range of tested particle sizes and hence provides a powerful tool for the in situ characterization of nanoparticle products. We hope that our work will add to the better understanding of TA-induced gold particle synthesis and provide a template for chemists to allow for more straightforward in situ characterization of particle products and hence support the more efficient determination of reaction conditions for nanomaterial synthesis.

## 2. Experimental Setup

### 2.1. Synthesis of the Gold Nanoparticles (GNPs)

Trisodium citrate (C_6_H_5_O_7_Na_3_H_2_O), tannic acid (C_76_H_52_O_46_), and hydrogen tetrachloroaurate trihydrate reagent (HAuCl_43_H_2_O) were purchased from Sigma-Aldrich. Deionized water was used throughout the whole procedure. The colloidal gold solutions (g1, g2, g3, and g4) were prepared according to the procedure reported by Slot and Geuze [17]. In order to prepare colloidal sample g1, typically 80 mL aqueous solution of HAuCl_4_ (2.5 × 10^−5^ mol) was heated to boiling. A 20 mL aqueous solution of tannic acid (2.9 × 10^−7^ mol) and trisodium citrate (15.5 × 10^−4^ mol) were also heated to boiling. The second solution was added to the HAuCl_4_ solution. After one hour, at the same temperature and with moderate stirring, the mix was refluxed during 2 min to get the red colored colloid. Similarly the remaining samples g2–g4 have been prepared, by varying the amount of the tannic acid and keeping the initial molarities of the sodium citrate and chloroauric constant. The final concentrations of TA were 2.9 × 10^−7^ mol (g1), 5.9 × 10^−7^ mol (g2), 5.9 × 10^−6^ mol (g3), and 4.1 × 10^−5^ mol (g4).

### 2.2. Instrumentation

Dynamic light scattering measurements were carried out using the instrument Zetasizer 3000 HSA, laser He-Ne (633 nm). Transmission electron microscopy (TEM) was performed using a JEOL 2000 EX electron microscope operated at 200 kV. Images were acquired using a Gatan Orius SC600 high-resolution camera.

## 3. Theory

### 3.1. Mie Theory

When the colloid is weakly or not aggregated, the Mie simulation process gives a very good agreement between the experimental and calculated spectra for both the shape and the intensity of the bands. The optical properties of gold nanospheres of diameter *D* were assessed in terms of their calculated absorption and scattering cross section (*C*_abs_ and *C*_sca_) and their localized surface plasmon resonance (LSPR). The main focus is to calculate the absorption efficiency since the scattering power is not significant for the nanoparticles with a size less than 40 nm [11]. *C*_abs_ is calculated using Mie exact theory (see (1) for homogeneous spheres) and the recurrence relations given by (2) were used to evaluate the Mie coefficients *a*_*n*_ and *b*_*n*_:

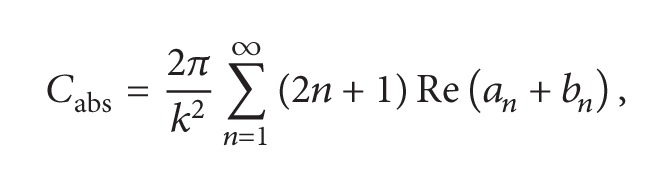
(1)

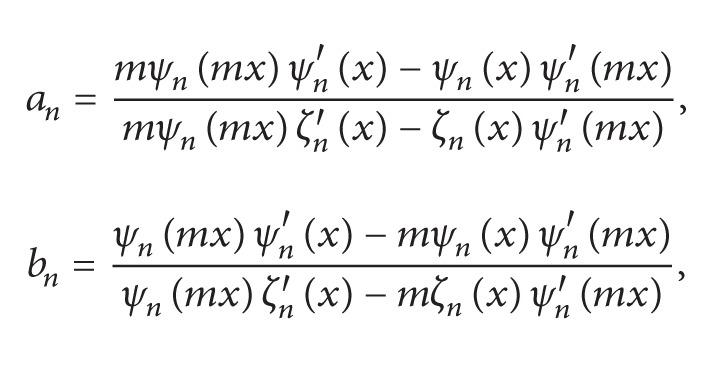
(2)
where *m* ^2^ is the ratio of dielectric function of the gold particles (the scatters) *ε*_*p*_ to that of the surrounding medium *ε*_sol_,  *k* is the wave-number of the light given by 2*πε*_sol_^1/2^/*λ*, with *λ* being the wavelength of the light in the vacuum, and *x* is the size parameter given as 2*πε*_sol_^1/2^(*D*/2)/*λ*. The refractive index of the surrounding medium (*n*_sol_) is related to the dielectric function by the following relation: *n*_sol_ = *ε*_sol_^1/2^ [15]. *ψ* and *ζ* are the Riccati-Bessel functions and the prime represents the first differentiation with respect to the argument in parentheses. Numerical calculations of the Mie series were performed at discrete points in the wavelength range from 300 to 700 nm. Computations of the optical absorption efficiency of GNPs were performed using a Bohren-Huffman computer code for concentric sphere geometry, adapted by B. T. Draine [4]. The required parameters for the code were the values of the GNP's diameter (*D*), the complex dielectric function of GNP (*ε*_*p*_), and the dielectric function of the surrounding medium (*ε*_sol_).

### 3.2. Dielectric Function of GNP

The major problem of the classical theories is the dependence of the optical constants on the wavelength of the light. This means that a complex dielectric function of the gold particles *ε*_*p*_ must be correctly described. We assume that this permittivity is similar to the one of the bulk metal. Taking into account the fact that the optical constants of the metal depend on the light frequency *ω* and according to the recent theory Drude-critical point called DCP model [23, 24], the gold dielectric function *ε*_*p*_ is given by the following expression:

(3) εp=ε∞−ωD2ω2+iγω +∑l=12AlΩleiϕlΩl−ω−iΓl+e−iϕlΩl+ω+iΓl.

In this equation, *ε*_*∞*_ is the metal permittivity at the higher frequencies, *ω*_*D*_ is a bulk plasma frequency, *γ* is the collision frequency, and the third term in the right-hand side of (3) represents the Lorentzian term. We report here the values of the various parameters calculated using DCP model: *ε*_*∞*_ = 1.1431, *ω*_*D*_ = 8.6638 eV, *γ* = 0.0709 eV, *A*_1_ = 0.2669, *ϕ*_1_ = −1.2371 eV, *Ω*_1_ = 2.5404 eV, Γ_1_ = 0.2929 eV, *A*_2_ = 3.0834, *ϕ*_2_ = −1.0968 eV, *Ω*_2_ = 2.7355 eV, and Γ_2_ = 1.5458 eV.

### 3.3. Dielectric Function of GNP Solution Prepared with TA

It is very common to use the effective medium rule of a two-phase composite to characterize the embedding medium as the aqueous solution. Here we consider components 1 and 2 as water and TA, respectively. The Bruggeman-Böttcher mixing rule [6, 7] given in (4) has been used to calculate the value of the representative dielectric function of the surrounding medium *ε*_sol_:

(4) εsol=14εw22−3Φ2+εTA23Φ−12hhhhhh+2εwεTA2+9Φ−9Φ21/2hhhhh+2−3Φεw+3Φ−1εTAεw22−3Φ2+εTA23Φ−12.

The term Φ in (4) represents the volume filling factor of TA in the aqueous solution. We have used the following values of the dielectric functions: for the water at optical frequencies *ε*_*w*_ = 1.77 and for TA *ε*_TA_ = 3.713 [28]. For simplicity, the contribution of the sodium citrate has been omitted in the calculation of the dielectric function of the surrounding medium.

## 4. Results and Discussion

**Figure 1:**
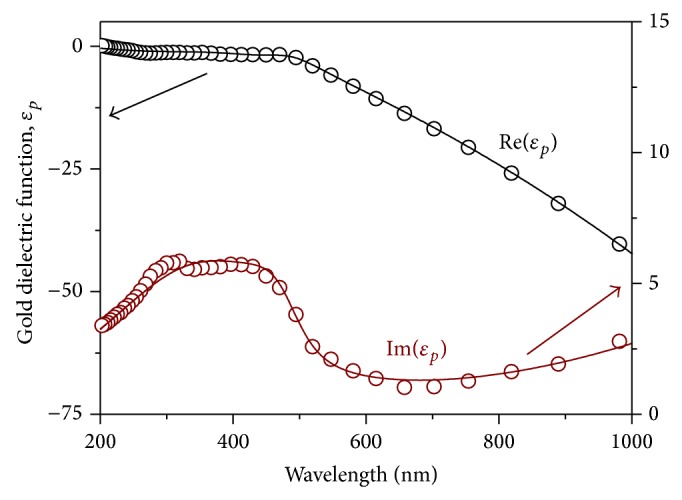
Complex gold dielectric (real and imaginary parts) as function of the light wavelength where the solid lines correspond to the DCP model and the symbols (○) represent the experimental data [15].

In order to elucidate and explore the effect of added TA on the optical properties of GNPs solutions, we calculated first the complex gold dielectric function (using the DCP model, see (3)) and compared it to the experimental data reported by Johnson and Christy [13]. The results are summarized in Figure 1 where a very good agreement between the theory and the experimental data is shown. One can see clearly that the model describes correctly the gold permittivity in a wide band of wavelengths ranging from 200 to 1000 nm.

**Figure 2:**
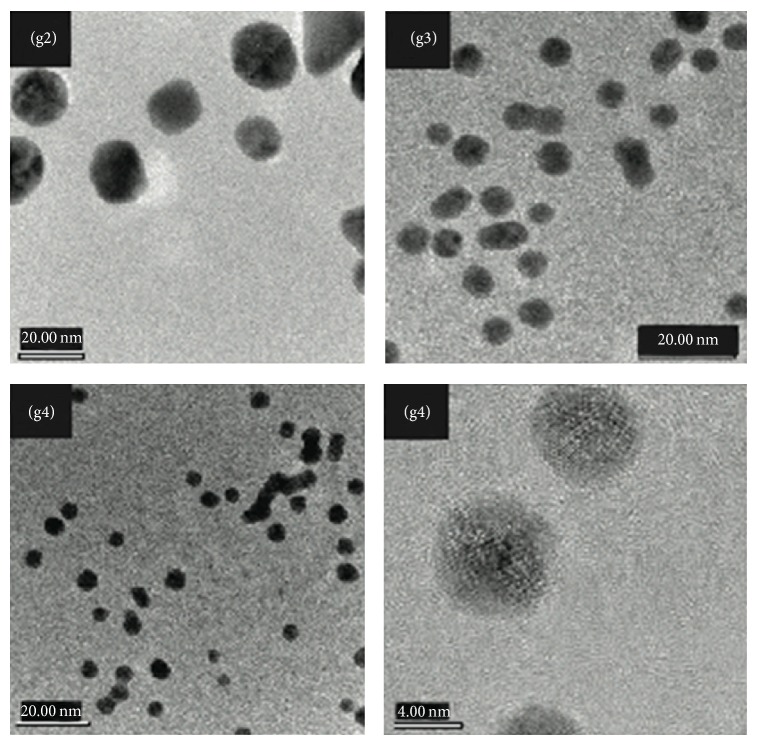
Transmission electron micrograph (TEM) of gold colloids g2, g3, and g4 synthesized in the presence of added TA with concentrations of 5.9 × 10^−7^, 5.9 × 10^−6^, and 4.1 × 10^−5^ mol, respectively.

The effect of synthetic conditions on the particle diameter was determined by both dynamic light scattering (DLS) and transmission electron microscopy (TEM). DLS analysis (performed using a MALVERN Zetasizer) yielded an average hydrodynamic diameter of gold particles equal to 26.6 nm (g1), 21.1 nm (g2), 7.8 nm (g3), and 4.2 nm (g4), respectively. These results are in excellent agreement with results obtained by electron imaging. For example, Figure 2 depicts representative bright field TEM micrographs of particle samples g2–g4 revealing both the particle size in agreement with DLS results and the crystalline nature of the particle systems.

**Figure 3:**
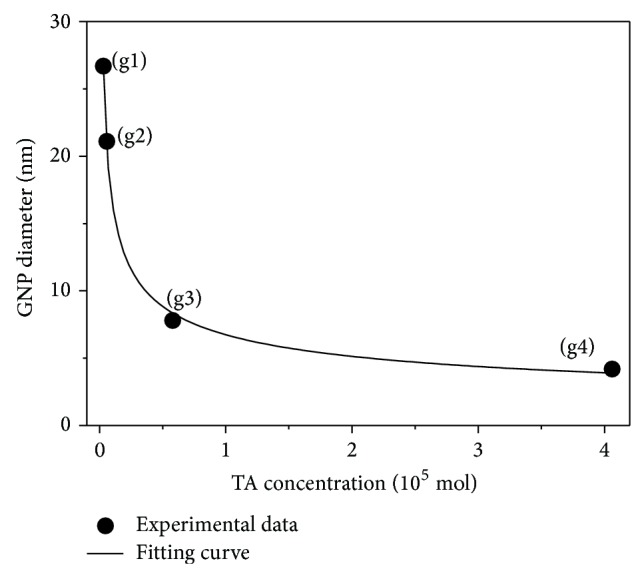
GNPs size as a function of added TA. The fitting curve corresponds to *y* = *ax* ^*b*^ with *a* = 0.074 and *b* = −0.39187.

In the following, DLS will be used as a reference for modeling analysis [29] since it averages over macroscopic volumes and hence large particle numbers.  Figure 3 summarizes the effect of TA concentration on the particle diameter along with a fitting curve *y* = *ax* ^*b*^ with *y* and *x* representing, respectively, the GNP size (nm) and the quantity of added TA (mol). The values of the parameters *a* and *b* corresponding to the best-fit curves are found to be 0.074 and −0.39187, respectively. The results show that as the quantity of tannic acid is increased, first the size decreases and then reaches saturation. Thus from these results it can be inferred that the amount of tannic acid plays an important role in the size dispersity of the gold nanoparticles.

Figure 3 shows also that tannic acid ceases to act as a reducing agent beyond the limit of 30 mL of TA (tannic to chloroauric acid ratio of 5.9 : 1). This pattern depends on the initial molarity of HAuCl_4_. According to [2], the authors concluded that, under varying initial gold chloride concentration, the size evolution of the generated GNPs exhibited two different patterns: one for HAuCl_4_ molarities less than 10^−3^ mol and the other one for the HAuCl_4_ molarities greater than or equal to 10^−3^ mol. When the molarity of HAuCl_4_ is less than 10^−3^ mol, the particle size continuously decreases with the increase in the tannic to chloroauric acid molar ratio. However, in the case where the HAuCl_4_ molarities are greater than or equal to 10^−3^ mol, a general increase in the size of the synthesized GNPs is observed even though the TA/HAuCl_4_ ratio increases; the saturation was achieved at tannic to chloroauric acid ratio of 9 : 1. In our study, the initial HAuCl_4_ concentration was smaller (2.5 × 10^−5^ mol).

**Figure 4:**
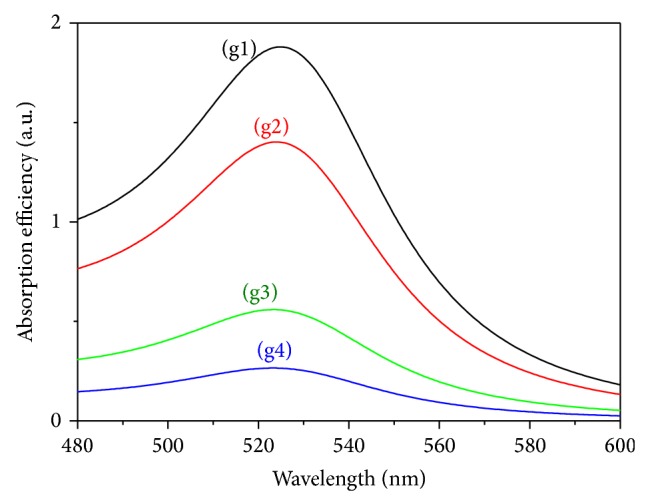
Absorbance efficiency of gold colloids (Mie theory) at different GNP size where the different curves (g1–g4) correspond, respectively, to 26.6, 21.1, 7.8, and 4.2 nm.

Figure 4 shows the calculated spectra of the absorption efficiency *Q*_abs_ for gold colloids as function of the wavelength and at different GNP size using Mie theory. The different curves (g1–g4) correspond, respectively, to *D* equal to 26.6, 21.1, 7.8, and 4.2 nm. The dimensionless efficiency *Q*_abs_ can be converted to the corresponding cross section *C*_abs_ by a simple multiplication with the cross-sectional area of the nanoparticle. The Mie simulation reveals that the peak of the absorption spectra decreases with decreasing GNP size. The narrow absorption band observed at 523 nm is the characteristic of almost spherical nanoparticles. For particles smaller than 2 nm, the LSPR diminishes completely as the energy levels for the electrons become discrete and instead step-like spectral transitions occur [8].

**Figure 5:**
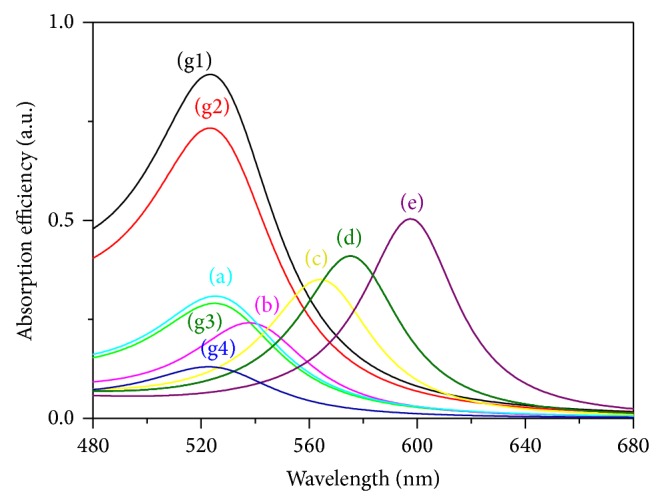
Absorbance efficiency (Mie theory) at different TA concentrations (10^5^ mol): g1 (0.029), g2 (0.059), g3 (0.59), g4 (4.1), a (22.2), b (50.0), c (60.0), d (80.0), and e (90.0).

Figure 5 depicts Mie simulation results for the case where the gold colloid solution is prepared with an increasing amount of the tannic acid. We observe first a reduction in particle size and therefore a decrease in the absorbance peak for the TA concentrations ranging from 0.029 × 10^−5^ mol to approximately 20 × 10^−5^ mol; then, upon reaching a certain amount of TA concentration, the peak absorbance begins to increase again. This band becomes more apparent for the particles with average diameters of 3 nm and TA molarity equal to 22.2 × 10^−5^ mol. The LSPR band becomes again broader and “red-shifts” to longer wavelength region (600 nm). The LSPR is affected by the refractive index of the ambient medium since the polarization of the particles will induce an opposing polarization in the ambient media. The liquid does not interact chemically with the surface but increases the refractive index, and a stepwise shift of the LSPR peak to longer wavelengths is observed. The addition of tannic acid to the GNPs solution does not lead to the particle aggregation but rather to a reduction in nanoparticle size from a mean diameter and gives a more convenient environmentally friendly method for the production of GNPs.

**Figure 6:**
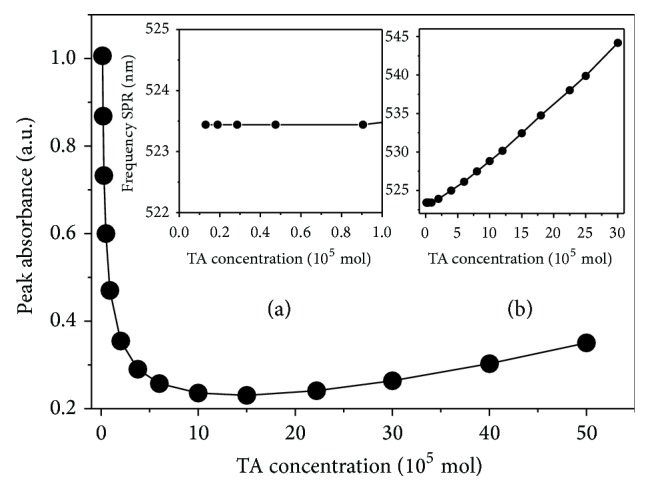
Peak absorbance as function of TA concentration. Inserts correspond to low concentration regime. (a) TA concentration less than 10^−5^ mol and (b) TA concentration up to 50 × 10^−5^ mol.

The decrease in the size leads to a decrease in the absorption as well as the relative scattering contribution. In Figure 6 we show the theoretical evolution of the peak absorbance and the plasmon frequency band revealing that such procedure is a good alternative to synthesizing small particles (smaller than 3 nm diameter) and having optical response in the infrared frequency.

## 5. Conclusion

We have presented a quantitative procedure to (in situ) analyze the size evolution of gold nanoparticles using Mie theory. Applied to the reduction of gold particles by tannic acid, the method reveals that the size evolution of gold nanoparticles sensitively depends on the concentration of added tannic acid. In particular, the particle size is found to decrease for a certain range of tannic acid concentrations. While we cannot provide a full interpretation of this effect, this finding demonstrates that, in tannic acid based synthesis, particle evolution depends on a more complicated completion of electrolytic reactions than previously reported. The theoretical study of the effect of TA concentration on the optical proprieties of GNPs colloid has shown that a shift in the surface plasmon resonance towards the nearly infrared frequencies occurs through a reduction in the GNPs size. We note that one benefit of the Mie model is its accuracy and applicability to arbitrary particle dimensions as compared to the more widely used procedures based on dipolar approximations. We therefore hope that our model will contribute to the better understanding of particle growth mechanisms.
